# RITA displays anti-tumor activity in medulloblastomas independent of *TP53* status

**DOI:** 10.18632/oncotarget.15840

**Published:** 2017-03-02

**Authors:** Aline Gottlieb, Kristina Althoff, Laura Grunewald, Theresa Thor, Andrea Odersky, Marc Schulte, Hedwig E. Deubzer, Lukas Heukamp, Angelika Eggert, Alexander Schramm, Johannes H. Schulte, Annette Künkele

**Affiliations:** ^1^ Department of Pediatric Oncology, University Hospital Essen, 45122 Essen, Germany; ^2^ Department of Pediatric Oncology, Hematology and SCT, Charité, 13353 Berlin, Germany; ^3^ Junior Neuroblastoma Research Group, Experimental and Clinical Research Center of the Max-Delbrück Center for Molecular Medicine (MDC), 13125 Berlin, Germany; ^4^ Institute for Pathology, University Hospital of Cologne, 50924 Cologne, Germany; ^5^ German Cancer Consortium (DKTK), 69120 Heidelberg, Germany; ^6^ German Cancer Research Center (DKFZ), 69120 Heidelberg, Germany; ^7^ Berlin Institute of Health (BIH), 10117 Berlin, Germany

**Keywords:** RITA, medulloblastoma, TP53, MDM2, CDKN1A

## Abstract

Current therapy of medulloblastoma, the most common malignant brain tumor of childhood, achieves 40–70% survival. Secondary chemotherapy resistance contributes to treatment failure, where TP53 pathway dysfunction plays a key role. MDM2 interaction with TP53 leads to its degradation. Reactivating TP53 functionality using small-molecule inhibitors, such as RITA, to disrupt TP53-MDM2 binding may have therapeutic potential. We show here that RITA decreased viability of all 4 analyzed medulloblastoma cell lines, regardless of TP53 functional status. The decrease in cell viability was accompanied in 3 of the 4 medulloblastoma cell lines by accumulation of TP53 protein in the cells and increased *CDKN1A* expression. RITA treatment in mouse models inhibited medulloblastoma xenograft tumor growth. These data demonstrate that RITA treatment reduces medulloblastoma cell viability in both *in vitro* and *in vivo* models, and acts independently of cellular TP53 status, identifying RITA as a potential therapeutic agent to treat medulloblastoma.

## INTRODUCTION

Medulloblastoma is the most common malignant brain tumor of childhood [1]. Despite multimodal therapy approaches including surgery, radiation and chemotherapy, overall survival of patients with medulloblastoma is currently 40–70% and this disease remains a major clinical challenge in pediatric oncology. Deficits in neurocognitive and neuroendocrine function, fertility, hearing and other long-term sequelae are common, and stem from aggressive treatment regimens [2–5]. Development of targeted approaches with fewer side effects is an essential step to improve long-term survival and quality of life in survivors. Since medulloblastomas are molecularly heterogeneous and studies have shown that molecular differences influence therapy response and outcome, therapy reduction should be considered for patients with tumor molecular profiles predicting good outcome as should therapy intensification for patients with profiles predicting poor outcome [6–8]. Four molecular subtypes designated as the WNT subtype, sonic hedgehog (SHH) subtype, Group 3 and Group 4 have been identified with different clinical outcomes [9]. While patients with medulloblastomas belonging to the WNT subtype have a 5-year overall survival of over 90%, 5-year overall survival in patients with a Group 3 medulloblastoma is only 40–60% [8, 10–12], underlining the importance of using this molecular subtyping to select the right therapy. Genetic alterations in the tumor also influence therapy response and outcome, including *MYCN* or *GLI2* amplifications or *TP53* mutations [13, 14]. Nevertheless, TP53 tumor suppressor dysfunction is rarely caused by the *TP53* mutations identified in medulloblastomas [15, 16], but mostly occurs in WNT and SHH medulloblastoma subtypes and relapsed medulloblastomas [17]. Our group has previously shown that overexpression of MDM2, leading to increased TP53 degradation, is frequently observed in medulloblastomas with wildtype *TP53*, and that nutlin-3, which inhibits TP53-MDM2 interaction, reduces tumor growth *in vitro* and *in vivo* [18]. Nutlin-3 treatment has also been shown to increase the frequency of *TP53* mutations in osteosarcomas and colon carcinomas, resulting in resistance [19]. Therefore, a treatment strategy to restore TP53 function in tumors harboring either wildtype or mutated *TP53* is required.

The RITA small molecule, whose name stems from reactivation of TP53 and induction of tumor cell apoptosis, binds to the N-terminus of TP53 and induces a conformational change that inhibits interaction with MDM2, resulting in anti-tumor activity *in vitro* and *in vivo* [20–23]. Interestingly, this effect was also seen in tumors harboring *TP53* mutations [24], questioning the original opinion that RITA works exclusively through blockade of the TP53-MDM2 pathway [20]. Here, we investigated whether RITA could reactivate the TP53 pathway in medulloblastomas independently of *TP53* mutational status, therefore, opening new avenues for the successful treatment of medulloblastomas, regardless of their *TP53* mutational status.

## RESULTS

### RITA reduces medulloblastoma cell viability *in vitro* independent of TP53 status

**Figure 1:**
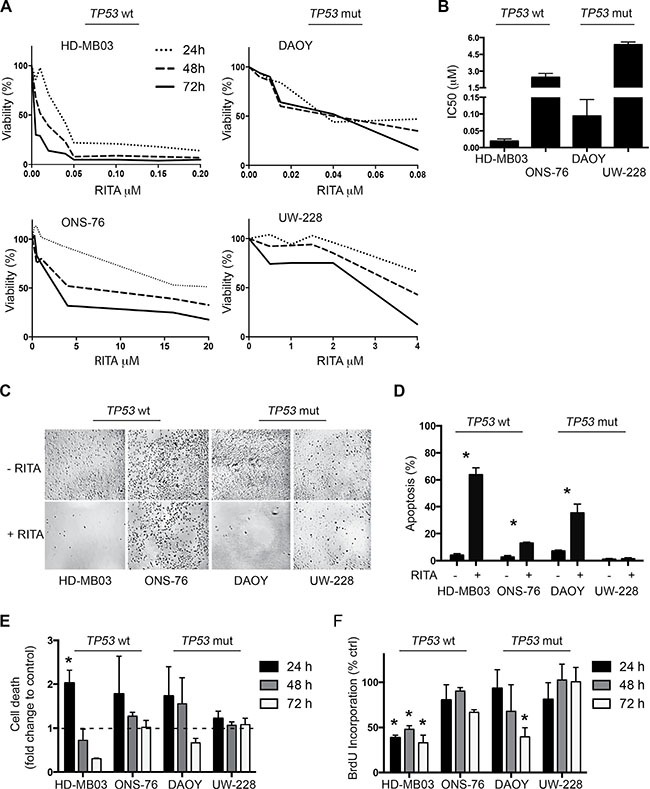
RITA treatment reduces cell viability and increases apoptosis in medulloblastoma cell lines with and without *TP53* mutations (**A**) Medulloblastoma cell viability was assessed using a 3-(4,5-dimethylthiazol-2-yl)-2,5-diphenyltetrazolinum bromide (MTT) assay in a time- and dose-dependent manner. (**B**) IC50 calculation of the data shown in A. (**C**) Microscopic pictures of the four adherent cell lines treated with 1 μM RITA for 48 h. Negative control: ethanol. (**D**) Assessment of apoptosis in medulloblastoma cell lines after 24 h of treatment with 1 μM RITA or ethanol by sub-G1 fraction FACS analysis. * = *P* < 0.05 in Student's *t-test*. (**E, F**) Cell death and proliferation analysis of medulloblastoma cell lines treated with 1 μM RITA for 24, 48 and 72 h using ELISA and BrdU Assay. Ethanol served as a negative control. * = *P* < 0.05 in Student's *t-test*.

We set out to explore the effect of RITA on medulloblastoma cell viability in culture and determine whether *TP53* mutational status alters RITA efficacy. We treated medulloblastoma cell lines harboring wildtype *TP53* (HD-MB03 and ONS-76) or *TP53* mutations (DAOY and UW-228-2) with varying concentrations of RITA for 24–72 h, then assessed cell viability. All 4 medulloblastoma cell lines displayed a reduction in cell viability during RITA treatment after 72 h, but the extent of reduction varied between the different cell lines (Figure 1A). Calculating the IC50s for the various medulloblastoma cell lines revealed that the anti-tumor effect of RITA was independent of *TP53* mutational status (*p* = 0.42; Figure 1B). While cells expressing wildtype TP53 displayed IC50s of 19.3 ± 6.4 nM (HD-MB03) and 2.5 ± 0.4 μM (ONS-76), cells harboring *TP53* mutations displayed IC50s of 94.0 ± 49.3 nM (DAOY) and 5.4 ± 0.2 μM (UW-228-2). The effect of RITA on the density of the adherently growing cell lines was also observed microscopically, where especially HD-MB03 and DAOY cultures were less dense after 48 h of RITA treatment compared to cells receiving only control medium containing the vehicle, ethanol (Figure 1C). We next examined whether RITA treatment could induce apoptosis in cultured medulloblastoma cell lines. We assessed the fraction of sub-G1 cells in cultures using FACS analysis to detect the apoptotic cell fraction. After 24 h of RITA treatment, the apoptotic fraction in HD-MB03 cultures was expanded by 16-fold (*P* = 0.004) compared to control cultures (Figure 1D). RITA treatment raised the apoptotic fraction in ONS-76 (*P* = 0.01) and DAOY (*P* = 0.03) cultures by 5-fold compared to controls, but had no significant effect on UW-228-2 apoptosis (Figure 1D). To confirm that RITA induced medulloblastoma cell death using an alternative method, we utilized an ELISA to detect mono- and oligonucleosomes in the cytoplasmatic fraction of cell lysates. RITA treatment induced cell death in HD-MB03 cells by 2-fold (*P* = 0.006) after only 24 h compared to untreated controls (Figure 1E). Data from ONS-76 and DAOY cell lines was too variable for apparent cell death increases to reach significance statistically, and RITA treatment had no significant effect on cell death in UW-228-2 cells in accordance with our data from the FACS analyses. We next investigated whether RITA treatment could halt medulloblastoma cell proliferation by assessing BrdU incorporation. HD-MB03 (*P* = 0.006) and DAOY (*P* < 0.0001) cell proliferation was dramatically reduced after 72 h of RITA treatment, whereas proliferation of ONS-76 and UW-228-2 cells remained unaffected (Figure 1F). Taken together, RITA demonstrated variable levels of anti-tumor activity against medulloblastoma cell lines, but variable efficacy did not correlate with the mutational status of TP53.

### RITA restores TP53 activity in medulloblastoma cell lines with and without TP53 mutations

**Figure 2:**
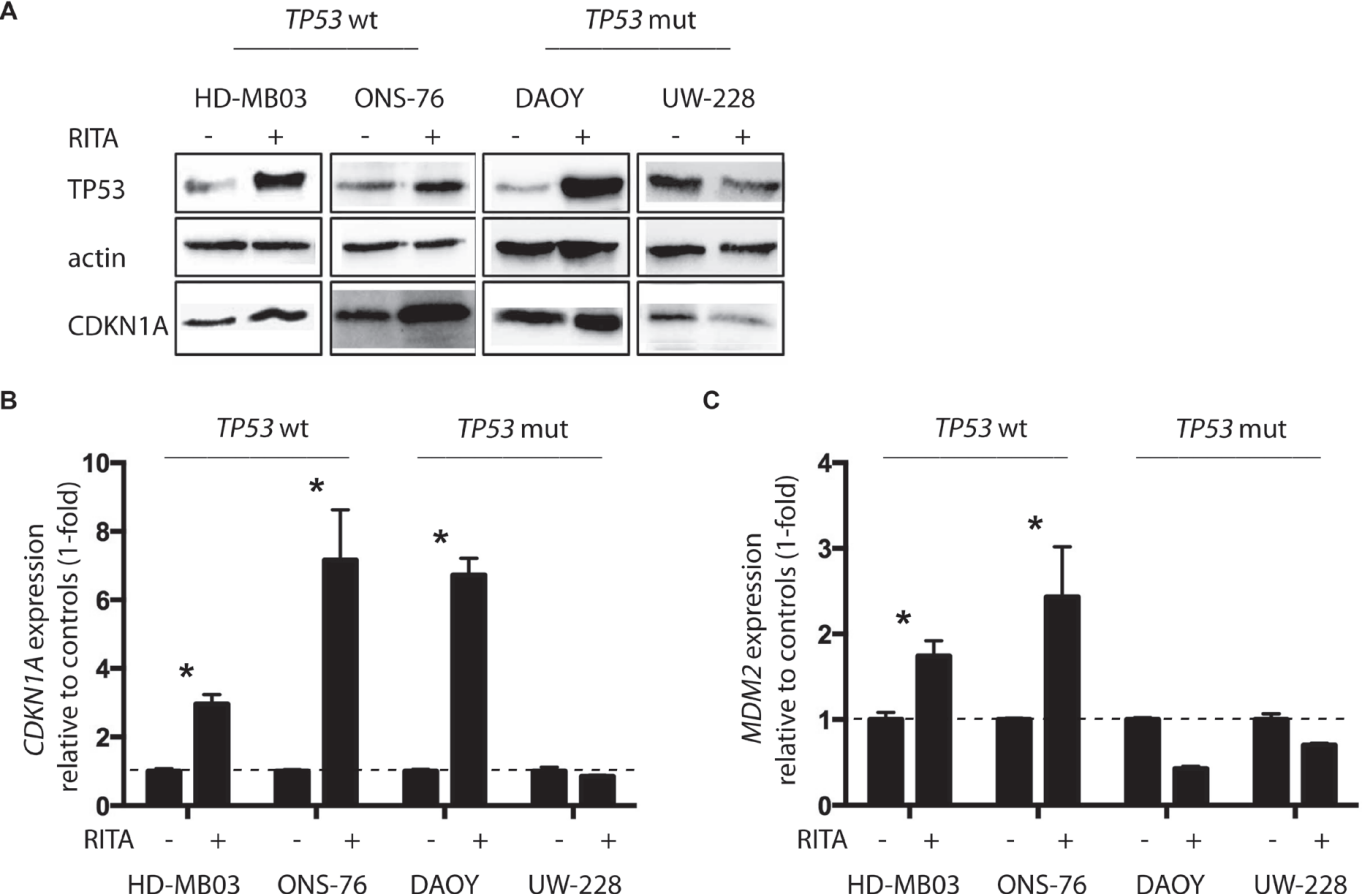
RITA restores *TP53* activity in medulloblastoma cell lines with and without *TP53* mutations (**A**) TP53 and CDKN1A protein expression after 48 h of RITA treatment assessed by western blot. Ethanol served as a negative control. wt = wildtype, mut = mutation. (**B, C**) Relative expression of *CDKN1A* and *MDM2* mRNA after 48 h of RITA treatment measured by real-time polymerase chain reaction (PCR). Ethanol served as a negative control. * = *P* < 0.05 in Student's *t-test*.

**Figure 3:**
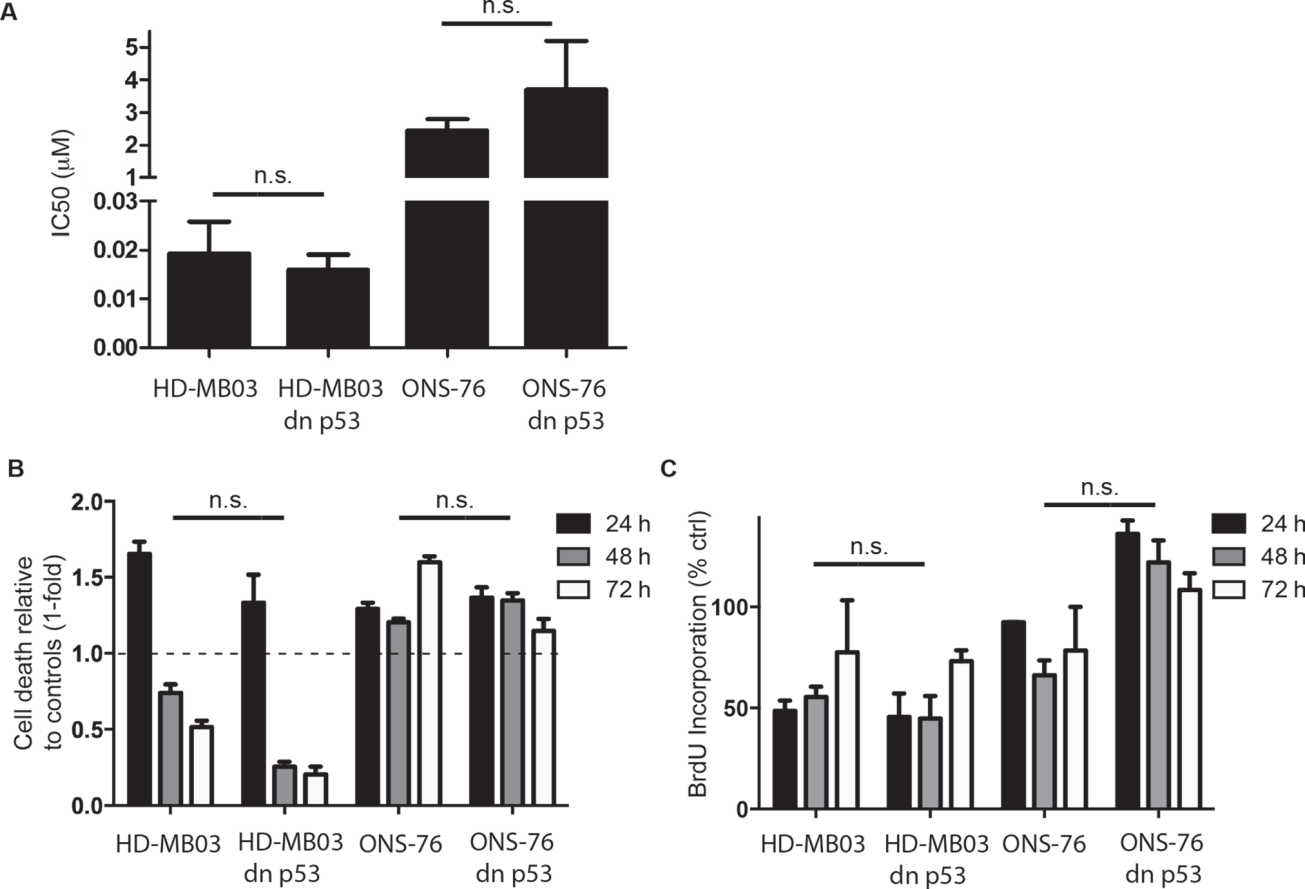
RITA reduces viability and proliferation in medulloblastoma cell lines expressing dominant-negative TP53 (dn-p53) (**A**) Determination of IC50s from HD-MB03 or ONS-76 with and without dn-p53 using cell viability data obtained by a 3-(4,5-dimethylthiazol-2-yl)-2,5-diphenyltetrazolinum bromide (MTT) assay. (**B, C**) Cell death and proliferation analysis of medulloblastoma cell lines treated with 1 μM RITA for 24, 48 and 72 h using ELISA and BrdU Assay. Ethanol served as a negative control.

To investigate whether RITA treatment reactivates TP53 signaling in medulloblastoma cells, we assessed expression of TP53 pathway components in the 4 medulloblastoma cell lines with and without RITA treatment. An increase in TP53 and CDKN1A expression was detected in HD-MB03, ONS-76 and DAOY cells after 48 h of RITA treatment (Figure 2A). Likewise, *CDKN1A* expression was also elevated in these 3 cell lines after RITA treatment (HD-MB03 3-fold higher, *P* = 0.003; ONS-76 7.2-fold higher, *P* = 0.002; DAOY 6.7-fold higher, *P* < 0.001; Figure 2B). Interestingly, RITA treatment induced *MDM2* expression only in cell lines with wildtype TP53 (HD-MB03 1.7-fold higher, *P* = 0.003; ONS-76 2.4-fold higher, *P* = 0.01; Figure 2C). We also enforced expression of a TP53 dominant negative mutant (dn-p53) in the medulloblastoma cell line most responsive to RITA treatment (HD-MB03) and the ONS-76 cell line, which was less sensitive to RITA treatment (Supplementary Figure 1). Enforced dn-p53 expression did not affect proliferation in either cell line (HD-MB03 wt vs HD-MB03 dn-p53 *p* = 0.47, ONS-76 wt vs ONS-76 dn-p53 *p* = 0.18; data not shown). We determined the IC50s for RITA treatment after dn-p53 expression compared to parental cell lines (Figure 3A). Enforced dn-p53 expression did not significantly influence the effect of RITA treatment, as measured by the IC50s (HD-MB03 vs HD-MB03 dn-p53, *P* = 0.07; ONS-76 vs ONS-76 dn-p53, *P* = 0.33), demonstrating that TP53 mutational status does not strongly impact the anti-tumor activity of RITA. We next examined whether enforced dn-p53 expression influenced the effect of RITA on apoptosis and cell proliferation. No differences in proliferation and cell death were detectable between transfected and non-transfected cell lines (Figure 3B, 3C), but increased CDKN1A was observed (Supplementary Figure 2). Taken together, our results support the hypothesis that RITA treatment activates TP53 signaling in medulloblastoma cell lines regardless of *TP53* mutational status.

### RITA treatment has anti-tumoral activity against human medulloblastoma cells grown as xenografts in mice

**Figure 4:**
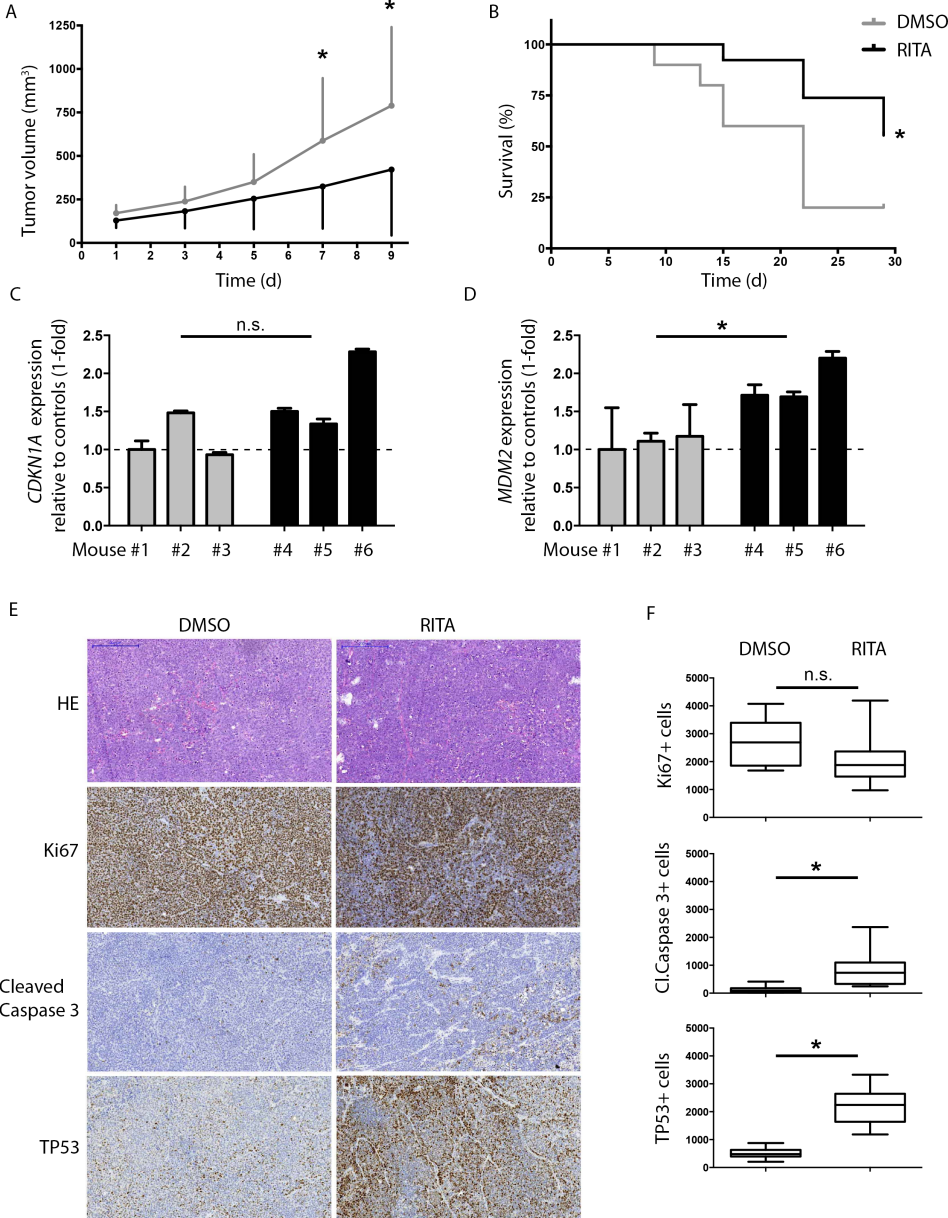
RITA treatment has anti-tumoral activity against medulloblastoma tumor xenografts in mice (**A**) Tumor growth response to intraperitoneal administration of RITA (*n* = 13), compared to DMSO (*n* = 10) in mice. * = *P* < 0.05 in Student's *t-test*. d = days. (**B**) Kaplan–Meier survival plot of treated cohorts from A. (**C, D**) Relative expression of *CDKN1A* and *MDM2* mRNA in tumors harvested after 3 days of DMSO (mouse#1, 2 and 3) or RITA (mouse#4, 5, 6) treatment. mRNA levels were measured using real-time polymerase chain reaction (PCR). Ethanol served as a negative control. * = *P* < 0.05 in Student's *t-test*. (**E, F**) Immunohistochemical analysis of proliferation (Ki67), apoptosis (cleaved caspase 3) and TP53 in tumors from mice treated for 3 days with RITA (*n* = 3) or DMSO (*n* = 3). * = *P* < 0.05 in Student's *t-test*.

To test the anti-tumoral activity of RITA in an *in vivo* model of medulloblastoma, we treated mice with established HD-MB03 xenografts once daily with RITA then assessed its efficacy. The treatment regimen was well tolerated, and did not cause weight loss or otherwise alter the physical status or behavior of the mice, or produce obvious signs of toxicity. The growth of HD-MB03 xenografts was significantly slowed in mice treated with RITA (*n* = 13) compared to mice treated with vehicle control (*n* = 10; Figure 4A). After 7 days of treatment, mean tumor volume progressed only to 324 mm^3^ in RITA-treated mice compared with the mean tumor volume of 588 mm^3^ for vehicle-treated mice (*P* = 0.04). With a primary end point defined as a tumor volume of 1000 mm^3^, the survival of the mice treated with RITA was significantly prolonged compared to mice treated with vehicle control (*P* = 0.04; Figure 4B). In a different, more intensive treatment model, RITA treatment was administered twice daily for 3 days and tumors were subsequently analyzed. Intensified RITA treatment did not increase *CDKN1A* expression, but increased *MDM2* expression (*P* = 0.01) and activated the TP53 pathway in the xenograft tumor tissue (Figure 4C–4F), which corresponded well with our results for RITA treatment of medulloblastoma cell lines grown *in vitro*. Immunohistochemical analysis of the xenograft tumors indicated RITA treatment increased apoptotic activity from increased levels of cleaved caspase 3 (*P* = 0.003, Figure 4E) and restored TP53 levels (*P* < 0.0001; Figure 4F). Thus, RITA activated the TP53 pathway *in vivo*, decreased xenograft tumor growth and increased mouse host survival.

## DISCUSSION

We report here that RITA shows anti-tumor activity *in vitro* against medulloblastoma cell lines independent of *TP53* status as well as *in vivo* against a xenograft tumor formed using a medulloblastoma cell line lacking *TP53* mutation. RITA exerted its anti-tumor effect and re-activated the TP53 pathway.

Reactivation of TP53 functionality has recently been shown to be achievable by different methods [15]. RITA, for example, binds to the MDM2 binding site of TP53, thereby blocking TP53 ubiquitination by MDM2. RITA has been demonstrated to have anti-tumor activity against cell lines derived from colon carcinoma, osteosarcoma, fibrosarcoma and myeloma [20, 25]. Importantly, even tumors that displayed resistance against standard chemotherapies remained sensitive to RITA treatment [26]. Here we demonstrated that RITA reduces cell viability and increases apoptosis of medulloblastoma cell lines. RITA treatment also induced transcript and protein expression of the TP53 downstream target, CKDN1A, demonstrating reactivation of TP53 function. Interestingly, *MDM2* expression was only induced in cell lines that did not harbor *TP53* mutations, indicating activation of the MDM2-TP53 feedback loop.

Existing *in vitro* data present some controversy about whether RITA requires intact TP53 activity to induce its anti-tumor effects. While earlier studies demonstrated that the anti-tumor effect was stronger in cell lines possessing wildtype TP53 than in cell lines harboring *TP53* mutations [26, 27], more recent publications showed RITA produced an anti-tumor effect independent of TP53 status [24, 25, 28]. Here, the lowest IC50 concentrations of RITA were obtained against the DAOY medulloblastoma cell line, which harbors a *TP53* mutation, and the HD-MB03 medulloblastoma cell line, harboring a non-functional SNP but otherwise wildtype *TP53*. RITA produced an anti-tumor effect in 3 of the 4 tested medulloblastoma cell lines, independent of their TP53 status. The IC50s obtained in our study were in the same range as those described by other groups. To further investigate whether sensitivity to RITA was dependent or independent of TP53 status, we enforced expression of a dominant-negative TP53 mutant. This did not abrogate the RITA effect, indicating that an intact TP53 pathway is not needed for medulloblastoma cell sensitivity to RITA. This is in line with results reported by Krajewski and colleagues, who demonstrated that RITA does not block the MDM2 binding site, suggesting RITA works independently of the TP53-MDM2 interaction [29]. One possible TP53-MDM2 pathway-independent anti-tumor effector mechanism could be the JNK or p38 pathway that was shown to be activated by RITA before [28, 30]. In summary, our data added further evidence for the hypothesis that RITA functions independently of the TP53 status.

Translation to the clinic for therapeutic use also requires *in vivo* evidence. Here we initially evaluated the efficacy of RITA treatment in a medulloblastoma xenograft mouse model using a dosage regimen previously shown to be safe for control mice and effective against neuroblastoma tumor xenografts [31]. RITA inhibited growth of medulloblastoma tumor xenografts and significantly prolonged mouse survival without causing visible signs of toxicity in the animals. These findings were consistent with previous studies of RITA in xenograft models of colon carcinoma, neuroblastoma, and mesothelioma [20, 22, 31, 32]. Interestingly, while RITA triggered *CDKN1A* and *MDM2* transcription in the HD-MB03 cell line, which harbors wildtype *TP53*, *in vitro*, only *MDM2* transcription was increased in xenograft tumors derived from HD-MB03 cells in mice receiving 3 days of the more intensive treatment in our study. Nevertheless, short-term RITA treatment was sufficient to increase TP53 expression and levels of cleaved caspase 3 in the xenografts, indicating activation of the TP53 pathway leading to apoptosis.

Our data support that the TP53 pathway can be re-activated by RITA treatment in medulloblastoma xenograft tumors, and that targeting the MDM2-TP53 axis using RITA may represent a potentially attractive therapeutic strategy for medulloblastomas independent of their *TP53* status. Medulloblastoma has a great biological heterogeneity as displayed in the currently used 4-subgroup classification defining risk and treatment strategy. Our data underline the importance of performing comprehensive genetic profiling early on during treatment and at relapse to detect different genetic variants, although it has been shown that the initial subgroup is maintained in relapse MB [33]. It is known that TP53 mutations occur in all 4 subgroups or in relapsed medulloblastoma that did not have a TP53 mutation at first diagnosis. Therefore patients can benefit from targeting the MDM2-TP53 axis independent of the subgroup the tumor belongs to. Our data underline that further molecular and cell surface markers should be used to define even more detailed risk group definitions helping to find the optimal therapy for the patient [34, 35]. RITA displayed promising anti-tumoral activity against medulloblastoma cells both *in vitro* and *in vivo*. RITA treatment prevented TP53 degradation by MDM2 in medulloblastoma cells effecting a re-activation of the TP53 pathway. In addition to this, RITA must also have reactivated TP53 activity independently of MDM2 and TP53 status, since RITA triggered *CDKN1A* transcription in medulloblastoma cells harboring either wildtype or mutated *TP53*. Ultimately, clinical testing of RITA in patients with medulloblastoma is necessary to demonstrate its usefulness therapeutically for the treatment of this highly aggressive pediatric malignancy. In light of the fact that about 30% of patients with medulloblastoma experience relapses and that relapse tumors are often resistant to chemotherapeutic agents, RITA might improve efficacy of standard relapse protocols as an additional component. Since the current treatment does not target the MDMD2-TP53 axis, RITA could provide another point of tumor attack to possibly improve overall survival in children with relapsed medulloblastoma.

## MATERIALS AND METHODS

### Cell lines and RITA treatment

The human medulloblastoma cell lines DAOY, HD-MB03, ONS-76, UW-228-2 (all adherently growing), were grown in RPMI 1640 (Lonza) supplemented with 10% fetal calf serum (GE Healthcare), penicillin (100 U/ml) and streptomycin (100 μg/ml; Life Technologies). Medium for HD-MB03 cells was additionally supplemented with 1% non-essential amino acids (Life Technologies) and 1% amphotericin B (GE Healthcare). Medium for ONS-76 cells was additionally supplemented with 1% L-glutamine (Life Technologies). All cell lines were authenticated by short tandem repeat DNA typing by the German Collection of Microorganisms and Cell cultures (Braunschweig, Germany) prior to the experiments. The genetic profile of the UW-228-2 cell line has not been previously described. Supplementary Table 1 lists all gene mutations detected in UW-228-2 and provided by the German Collection of Microorganisms and Cell cultures. RITA (Cayman Chemicals) was dissolved in ethanol and stored as a 10 mmol/L stock solution at −20°C. Cells were exposed to 0–8 μM RITA for the period indicated, with the final ethanol concentration kept constant in each experiment. For rescue experiments, HD-MB03 and ONS-76 cells were transfected with an expression construct for dominant negative TP53 (donated by Prof. M. Eilers) [36]. Selection of transfected cells was achieved by addition of puromycin (1 g/ml; Life Technologies) to the medium.

### Western blot analysis

Protein lysates were extracted from cells treated with 1 μM RITA for 24, 48, and 72 h, lysed 30 min on ice in radioimmunoprecipitation assay (RIPA) Buffer supplemented with Complete Protease Inhibitor Cocktail Tablets and PhosSTOP Phosphatase Inhibitor Cocktail Tablets (Roche). 50 μg proteins were separated on 10% SDS-PAGE then transferred to Amersham-Hybond™-C Extra (GE Healthcare) membranes. Transferred membranes were blocked in 5% milk powder in Tris-buffered saline and Tween 20 and then incubated for 12 to 24 h, using the following antibodies and dilutions: CDKN1A (1:500, cat ab-7960, Abcam), TP53 (1:500, cat#sc-71817, Santa Cruz Biotechnology), MDM2 (1:1000, IF2, cat#33-7100, Life Technologies), beta-actin (1:2000, cat#A5541, Sigma-Aldrich), GAPDH (1:2000, cat#MAB374, Merck Millipore), and HRP-conjugated anti-rabbit IgG (1:2000; GE Healthcare) or HRP-conjugated anti-mouse IgG (1:2000; GE Healtcare) was added for 1 h at room temperature. Proteins were visualized using Amersham ECL Plus™ western blotting detection reagents (GE Healthcare) and the UVchem Detection Device (Biometra).

### Gene expression analysis

*TP53, MDM2* and *CDKN1A* expression was monitored using real-time polymerase chain reaction (PCR) using Assays on Demand (Applied Biosystems-Life Technologies). Expression values were normalized to the geometric mean of *GAPDH*. For all these experiments, total RNA was isolated from cells with use of the RNeasyMini kit (Qiagen) and cDNA synthesis was performed using the SuperScript^®^ reverse transcription kit (Life Technologies).

*dnP53* was measured using the following primer: 5′GGAGCACTAAGCGAGCACTG3′ (sen) and 5′-TATGG CGGGAGGTAGACTGA-3′ (rev) and normalized to endogenous P53 expression.

### Cell viability, proliferation and cycle analysis

Cells were seeded onto 96-well plates (5– 7.5 × 10^3^ per well) in triplicate, incubated for 6 h to permit surface adherence, and treated with 1 μM RITA for 24, 48 and 72 h. Medium was replaced daily, RITA and ethanol concentrations were constant throughout the experiment. Cell viability was analyzed using the 3-(4,5-dimethylthiazol-2-yl)-2,5-diphenyltetrazolinum bromide (MTT) assay (Roche) and IC50 was calculated using GraphPad Prism 5.0 (GraphPad Incorporation). Apoptosis was assessed using the Cell Death ELISA (Roche). Cell proliferation was determined using the Bromdesoxyuridin (BrdU) ELISA (Roche). All three assays were performed according to the manufacturer's protocol. For cell cycle analysis, cells were incubated for 15 min with propidium iodide (10 μg/ml) and cellular content was analyzed using a FC500 flow cytometer (Beckman Coulter).

### Immunohistochemistry

Xenograft tumors were formalin fixed, paraffin embedded, and 5 μm sections were cut from each block. Immunohistochemical staining was conducted as described before [18]. Primary antibodies used were: TP53 (1:250, cat#ab4060, Abcam), Ki67 (1:500, cat#ab16667, Abcam) and cleaved caspase 3 (1:200, cat#9661, Cell Signaling Technology). The slides were developed using the EnVision (Dako).

### Growth of xenograft tumors in nude mice

HD-MB03 medulloblastoma cells were cultured to 80% confluence, harvested, and suspended in 200 μL Matrigel (BD Biosciences) for subcutaneous inoculation (1.5 × 10^7^ cells per mouse) into the left flank of 8-week-old female athymic NCR (nu/nu) mice. After reaching a tumor size between 100 and 300 mm^3^, mice were (randomly) assigned to two groups. The first group was the standard treatment group in which the mice were treated intraperitoneally either with RITA (0.3 mg/mouse/day) or with a vehicle control over multiple days in order to analyze the effect of RITA on tumor growth and survival of the mice. The second group of mice received for 3 days an extensive i.p. treatment of RITA twice daily (2 × 0.6 mg/mouse/day) or the vehicle control. In this group the mice were euthanized after the 3-day treatment period and tumors were harvested for immunohistochemical and gene expression analysis to investigate the immediate effect of RITA on the tumor. Each group was further randomly assigned to either RITA or vehicle control groups. RITA or vehicle control was administered with 10% Dimethylsulfoxid (DMSO) in PBS (Gibco^®^, Life Technologies). Tumor growth was monitored daily using a caliper, and tumor volume was calculated using the formula (breadth × length × height)/2. Mice were euthanized by cervical dislocation when tumor size exceeded 1000 mm^3^. The tumor was removed and a third was formalin fixed and paraffin embedded, the other two thirds were snapped frozen in liquid nitrogen and then stored at −80°C. The mice receiving intensive treatment were euthanized 12 h after the last injection. All experiments were performed in accordance with the Council of Europe guidelines for accommodation and care of laboratory animals and protocols were approved (reference number 87–51.04.2010.A158) by the North Rhine-Westphalia State Environment Agency (Landesamt für Natur, Umwelt und Verbraucherschutz NRW).

### Statistical analysis

Graph Pad Prism was used to conduct statistical analysis, calculate IC50 concentrations, design graphs, and perform Kaplan-Meier survival analysis with log-rank statistics for the mouse cohorts. Data are presented as means ± SD. The Student *t* test was conducted as a two-sided unpaired test with a confidence interval of 95%. Results with a *P value* of less than 0.05 were considered statistically significant. *MDM2* and *CDKN1A* mRNA expression levels were calculated using biogazelle (www.biogazelle.com). Ki67, cleaved caspase 3 and TP53 protein expression was assessed analyzing 5 immunohistochemically stained slides of each of the tumors using the ImageJ program [37].

## SUPPLEMENTARY MATERIALS FIGURES AND TABLES


