# Supplementary material for: RITA displays anti-tumor activity in medulloblastomas independent of TP53 status
Source: Oncotarget. 2017 Mar 2;8(17):27882–91. doi: 10.18632/oncotarget.15840 (PMC5438615; doi:10.18632/oncotarget.15840)
Supplement: Supplementary file 1 [file oncotarget-08-27882-s001.pdf]

RITA displays anti-tumor activity in medulloblastomas independent of *TP53* status

Supplementary Materials

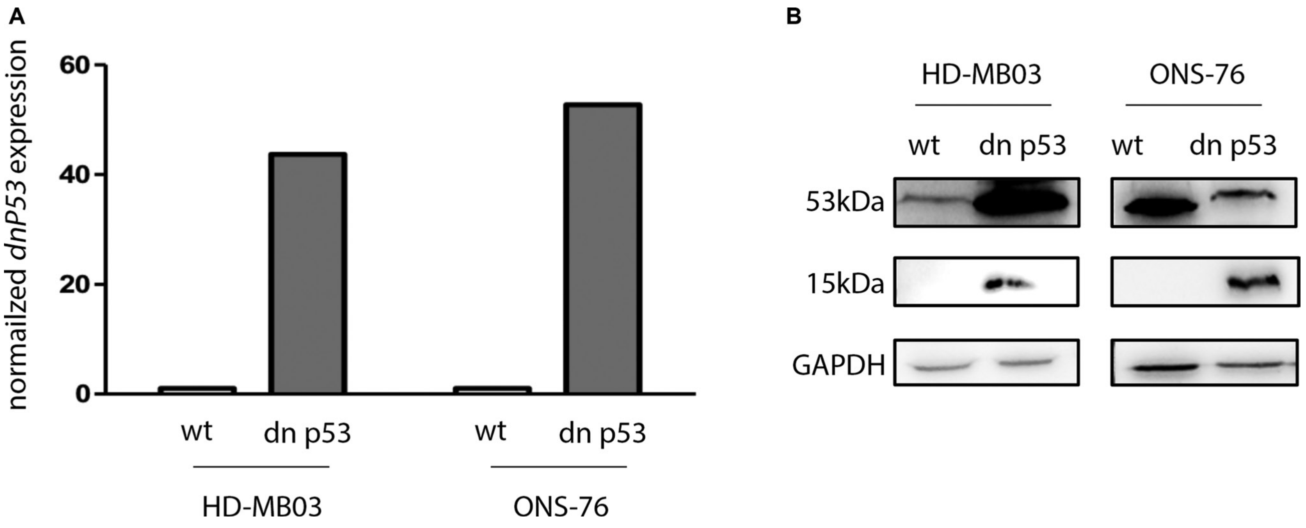

Supplementary Figure 1: The medulloblastoma cell lines HD-MB03 and ONS-76 express dominant-negative TP53 (dn-p53). (**A**) Levels of the dn-p53 were measured using quantitative polymerase chain reaction (qPCR) and normalized to levels of endogenous *TP53*. (**B**) Protein expression of TP53 and its truncated dn-p53 detected by western blot. wt = wildtype, dn = dominant negative.

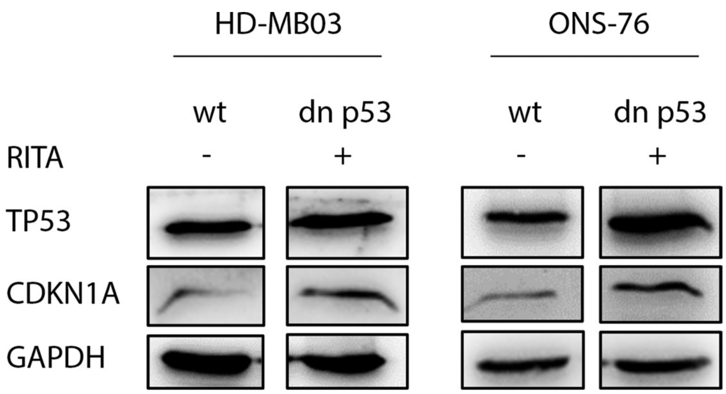

Supplementary Figure 2: RITA treatment re-activates the *TP53* pathway in HD-MB03 and ONS-76 expressing the dominant-negative TP53 (dn-p53). TP53 and CDKN1A protein expression after 72 h of RITA treatment is assessed by western blot. Ethanol served as a negative control. wt = wildtype, dn = dominant negative.

Supplementary Table 1: Genetic profile of UW-228-2

Marker Name	Allele Size
AMEL	X
CSF1PO	11, 13
D13S317	13
D16S539	11
D18S51	16
D21S11	28, 31
D3S1358	17
D5S818	11, 12
D7S820	11
D8S1179	12, 13
FGA	22
Penta D	9, 12
Penta E	17
TH01	9.3
TPOX	11
vWA	14
